# First Account of Primary Leiomyosarcoma of the Lesser Omentum since Establishment of c-Kit Mutations in Gastrointestinal Stromal Tumors

**DOI:** 10.1155/2019/2426092

**Published:** 2019-11-04

**Authors:** Fumito Saijo, Kaoru Sato, Tomoko Handa, Yoichi Narushima, Naoki Matsumura, Noriyuki Iwama, Fumie Nakayama, Hiromi Tokumura

**Affiliations:** ^1^Department of Surgery, Tohoku Rosai Hospital, 4-3-21, Dainohara, Aobaku, Sendai, Miyagi 981-8563, Japan; ^2^Department of Gastroenterology, Tohoku Rosai Hospital, 4-3-21, Dainohara, Aobaku, Sendai, Miyagi 981-8563, Japan; ^3^Department of Pathology, Tohoku Rosai Hospital, 4-3-21, Dainohara, Aobaku, Sendai, Miyagi 981-8563, Japan

## Abstract

**Background:**

Primary omental leiomyosarcoma is an extremely rare type of tumor. Leiomyosarcomas originating from the lesser omentum have not been reported since immunohistochemical staining for c-kit has been used for the diagnosis of mesenchymal abdominal tumors. They are yet to be reported since gastrointestinal stromal tumors were categorized. Here we reported a case of successful resection of a lesser omental leiomyosarcoma.

**Case Presentation:**

A 71-year-old man underwent ultrasonography at the outpatient clinic through which an upper abdominal tumor was identified. Following computed tomography and endoscopy, a 4.5 cm submucosal tumor in the lesser curvature of the stomach was highly suspected. A laparoscopic partial resection of the stomach was performed. Histopathological examination revealed the tumor to be a leiomyosarcoma arising from the lesser omentum that did not invade the stomach. Immunohistochemical staining showed that the tumor was negative for CD34, c-kit, and S-100 and positive for desmin and *α*-smooth muscle actin. No recurrence had been observed 1 year after surgery without adjuvant chemotherapy.

**Conclusions:**

Primary lesser omental leiomyosarcoma, which is difficult to diagnose before surgery given the location of the primary tumor in the lesser omentum, has rarely been reported. Considering the high possibility of malignancy, close observation is essential.

## 1. Introduction

Abdominal leiomyosarcomas have rarely been reported since Hirota et al. first described gastrointestinal stromal tumors (GISTs) with c-kit mutations [1]. Primary lesser omental tumors have also been infrequently identified. They arise most often from the uterus, gastrointestinal tract, and retroperitoneal region. Here we described the first case of lesser omental leiomyosarcoma since c-kit mutations in GISTs had been established.

## 2. Case Presentation

**Figure 1:**
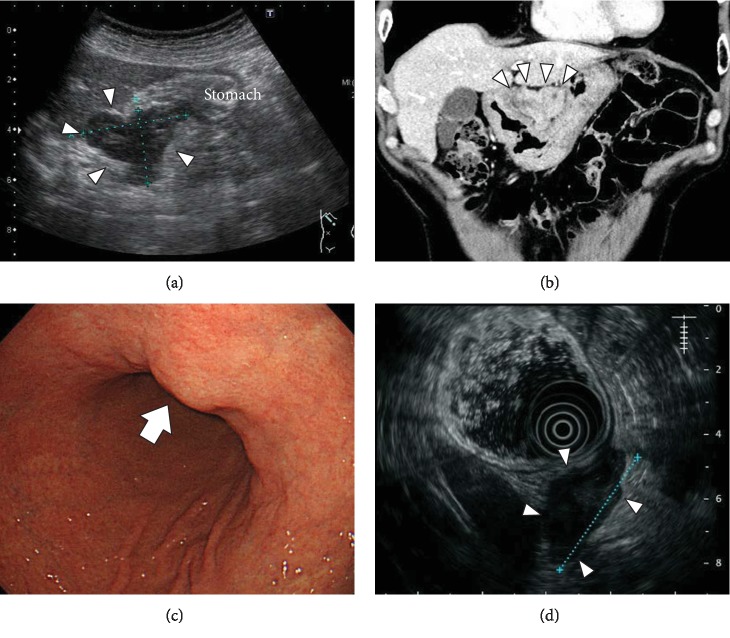
Preoperative imaging findings. Abdominal ultrasonography (AUS) showing a leaf-shaped tumor (arrowhead) with a mosaic pattern. (a) Computed tomography (CT) showing internal heterogeneity (arrowhead) between the head portion of the pancreas and the left liver lobe, (b) upper gastrointestinal endoscopy showing an elevated, nonepithelial lesion with a smooth surface in the lesser curvature of the stomach (arrow), (c) endoscopic ultrasonography (EUS) showing the same tumor (arrowhead) that seemed to be continuous with the fourth layer, and (d) the tumor was approximately 49 × 29 mm^2^ in size. The tumor mass appeared to protrude from the stomach wall.

A 71-year-old man, who did not complain of any physical pain, decided to consult our hospital's hepatology department owing to outpatient laboratory data and ultrasonography results that suggested slight liver dysfunction and an upper abdominal tumor, respectively. The patient's medical history included appendectomy at the age of 16 years, right thigh fracture at the age of 22 years, and hyperlipidemia since he was 60 years old. Laboratory data showed aspartate aminotransferase, alanine transaminase, and alkaline phosphatase levels of 28, 43, and 469 IU/L, respectively. However, following abdominal ultrasonography (AUS) (Figure 1(a)), computed tomography (CT) (Figure 1(b)), and upper gastrointestinal endoscopy, a 4.5 cm submucosal tumor at the lesser curvature of the stomach was highly suspected. Upper gastrointestinal endoscopy showed an elevated, nonepithelial lesion with a smooth surface in the lesser curvature of the stomach (Figure 1(c)). AUS revealed a leaf-shaped tumor with a mosaic pattern and a heterogeneous parenchyma. Moreover, endoscopic ultrasonography (EUS) (Figure 1(d)) showed that the same tumor seemed to be continuous with the fourth layer, and contrast-enhanced CT revealed that the heterogeneous parenchyma was located between the head portion of the pancreas and the left liver lobe. The size of the tumor was approximately 49 × 29 mm. The tumor mass appeared to protrude from the stomach wall (Figures 1(a)–1(d)). AUS and CT did not indicate liver tumor, lymph node swelling, and peritoneal dissemination. The preoperative diagnosis was submucosal tumor, a suspected GIST of the stomach.

**Figure 2:**
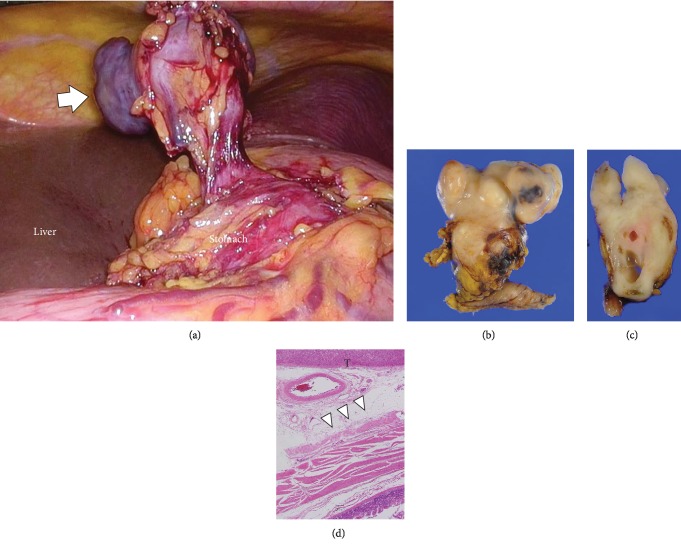
Operative findings and macrospecimen. As the lesser omentum and fat around the tumor (arrow) were exfoliated, the tumor appeared to be continuous with only a portion of the stomach wall (a). Histopathologic examination revealed a tumor with a multinodular but smooth outer surface and foci of fleshy and pale cream-yellow areas underneath. The excised specimen after application with formalin (b) and the cut surface of the resected specimen (c), and pathological findings showed that the tumor (T) was not continuous with the stomach serosa (arrowhead) (d).

Laparoscopic partial resection of the stomach was subsequently performed. Although the tumor was located at the lesser curvature of the stomach, its origin could not be determined by surgical findings alone. As the lesser omentum and fat around the tumor were exfoliated, the tumor appeared to be continuous with only a portion of the stomach wall. The tumor, as well as a portion of the stomach, was completely resected, including the stomach wall believed to be continuous with the tumor (Figure 2(a)). The procedure time was 65 min with no blood loss. Length of stay was 8 days after operation. No complication was detected.

**Figure 3:**
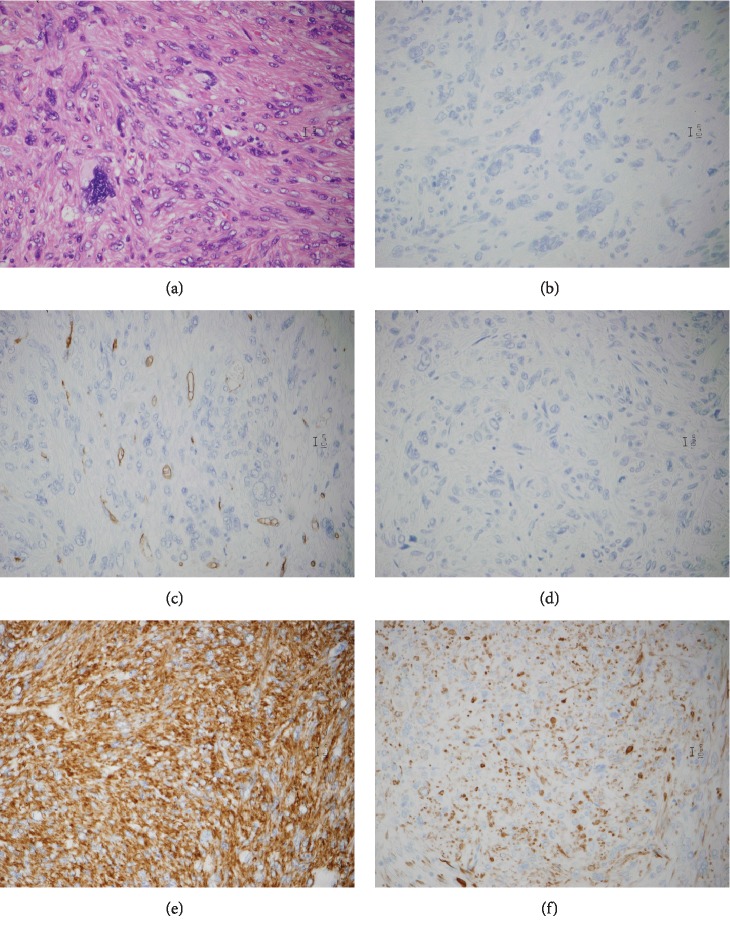
Microscopic examination. Microscopic examination confirmed the diagnosis of leiomyosarcoma with long intersecting fascicles of spindle cells (hematoxylin and eosin staining) (a). Immunohistochemical staining revealed that the tumor was negative for c-kit (b), CD34 (c), S-100 (d), and DOG1 and positive for *α*-SMA (e), desmin (f), HHF-35, h-caldesmon, and calponin.

A diagnosis of leiomyosarcoma was established. Histopathologic examination of the tumor revealed a multinodular but smooth outer surface and foci of fleshy and pale cream-yellow areas underneath (Figures 2(b) and 2(c)). Pathological findings showed that the tumor was not continuous with the stomach serosa (Figure 2(d)). Moreover, microscopic examination revealed long intersecting fascicles of spindle cells (hematoxylin and eosin staining), confirming the diagnosis of leiomyosarcoma (Figure 3(a)). Immunohistochemical staining showed that the tumor was negative for c-kit (Figure 3(b)), CD34 (Figure 3(c)), S-100 (Figure 3(d)), and DOG1 and positive for *α*-smooth muscle actin (*α*-SMA) (Figure 3(e)), desmin (Figure 3(f)), HHF-35, h-caldesmon, and calponin. High Ki-67 (MIB-1) expression (55.4%) and mitotic rate (39/50HPF) were also observed.

After providing informed consent that there are no established drugs as postoperative adjuvant chemotherapy, and at 6 months and 1 year after surgery, no signs of recurrence were detected by CT.

## 3. Discussion

Leiomyosarcomas comprise approximately 5%–10% of all soft-tissue sarcomas [2]. They arise most often from the uterus, gastrointestinal tract, and retroperitoneal region [3]. Abdominal leiomyosarcomas have been rarely reported since Hirota et al. first described GISTs with c-kit mutations [1]. One such study is that by Barel et al. who reported a case involving primary leiomyosarcoma of the omentum and reviewed 27 other cases previously published in the literature [4]. Moreover, lesser omental leiomyosarcomas are even more infrequent with only three cases being identified using a PubMed search with the keywords “lesser omentum” and “leiomyosarcoma.” However, all three reports did not include c-kit immunostaining, which would help distinguish leiomyosarcoma from GIST [5–7]. The following omental tumors have also been reported: extra-GIST, leiomyosarcoma, fibrosarcoma, hemangiopericytoma, spindle cell sarcoma, liposarcoma, leiomyoma, lipoma, desmoid tumor, fibroma, and mesothelioma [8–12].

Although slight liver dysfunction prompted AUS in the present case, it had no correlation with the tumor given the spontaneous improvement in liver function. AUS and CT allowed for the detection of the tumor and accurate identification of its internal structure. EUS also indicated the presence of a tumor, which appeared to be continuous with the stomach wall. However, the EUS findings that the tumor was continuous with the stomach wall were misjudged more than pathological findings. Hence, GIST was ultimately suspected before surgical treatment. Given the lack of equipment at our hospital, EUS-guided fine-needle aspiration biopsy (EUS-FNAB) could not be performed. According to the Japanese GIST guideline, small submucosal tumors (SMTs) 2–5 cm in size should preferably be diagnosed using EUS-FNAB albeit not required [13]. Furthermore, the treatment strategy indicated in this guideline suggests that malignant findings during CT and EUS can be an indication for surgery.

Leiomyosarcoma can be pathologically distinguished from GIST and schwannoma. Accordingly, a GIST is characterized by c-kit(+)/desmin(−)/S-100(−), leiomyoma by c-kit(−)/desmin(+)/S-100(−), and schwannoma by c-kit(−)/desmin(−)/S-100(+). Although the positivity rate for CD34 in GISTs is approximately 91%, which is lower than that for c-kit, it is very rarely positive in gastrointestinal mesenchymal tumors other than GISTs [14]. DOG1 also has a positivity rate of 97% in GISTs and can be used for diagnosis [15]. Moreover, considering that *α*-SMA is almost 100% positive for smooth muscle tumors, around 31% positive for GISTs, and negative for other tumors, it may have utility in distinguishing GISTs from sarcomas [14]. Similar to desmin, HHF-35 is a myogenic marker that is positive in both smooth and striated muscle tumors, whereas h-caldesmon and calponin are positive in smooth muscle tumors. Our immunohistochemical analysis showed that the tumor was c-kit(−), CD34(−), and desmin(+), which led us toward the diagnosis of leiomyosarcoma.

Complete surgical resection has been the best treatment option for leiomyosarcoma. Given a preoperative diagnosis of gastric SMT in the present case, local gastric resection had been performed according to the guidelines for tumor resection [13]. Surgery is required to ensure a surgical safety margin without damaging the capsule, leaving a gross stump that tests negative [16]. In a summary of 27 reports of primary omental leiomyosarcoma by Barel et al., 11 cases died during follow-up. Although the true prognosis for lesser omental leiomyosarcoma is unknown, it is likely to be poor [4]. Although the apparent prognosis for lesser omental leiomyosarcoma has remained unknown, the possibility of poor prognosis has been suggested.

Given the rarity of primary lesser omental leiomyosarcomas, we believe that reporting such cases is important. Moreover, it is difficult to determine whether the primary tumor originates from the lesser omentum before surgery. Although lesser omental leiomyosarcomas are rare, their malignant potential may be high, thereby necessitating close observation.
